# 1-Chloro­acetyl-3,3-dimethyl-2,6-di­phenyl­piperidin-4-one

**DOI:** 10.1107/S1600536808030985

**Published:** 2008-09-30

**Authors:** T. Kavitha, S. Ponnuswamy, M. Jamesh, J. Umamaheshwari, M. N. Ponnuswamy

**Affiliations:** a Centre of Advanced Study in Crystallography and Biophysics, University of Madras, Guindy Campus, Chennai 600 025, India; b Department of Chemistry, Government Arts College (Autonomous), Coimbatore 641 018, India

## Abstract

In the mol­ecule of the title compound, C_21_H_22_ClNO_2_, the piperidine ring adopts a distorted boat conformation. The two phenyl rings are nearly orthogonal to each other with a dihedral angle of 87.1 (1)°. In the crystal structure, the mol­ecules are linked into a three-dimensional network by C—H⋯O and C—H⋯π inter­actions.

## Related literature

For general background, see: Dimmock *et al.* (2001 ▶); Perumal *et al.* (2001 ▶). For ring conformational analysis, see: Cremer & Pople (1975 ▶); Nardelli (1983 ▶).
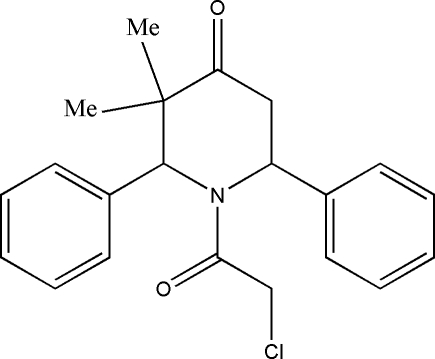


## Experimental

### 

#### Crystal data

C_21_H_22_ClNO_2_
*M* *_r_* = 355.85
Monoclinic, 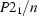

*a* = 13.7005 (3) Å
*b* = 9.8735 (2) Å
*c* = 14.8960 (3) Å
β = 112.762 (1)°
*V* = 1858.08 (7) Å^3^
*Z* = 4
Mo *K*α radiation
μ = 0.22 mm^−1^
*T* = 293 (2) K
0.32 × 0.26 × 0.20 mm

#### Data collection

Bruker Kappa APEXII area-detector diffractometer
Absorption correction: multi-scan (*SADABS*; Sheldrick, 2001 ▶) *T*_min_ = 0.854, *T*_max_ = 0.958
22063 measured reflections
4674 independent reflections
3424 reflections with *I* > 2σ(*I*)
*R*_int_ = 0.025

#### Refinement

*R*[*F* ^2^ > 2σ(*F* ^2^)] = 0.050
*wR*(*F* ^2^) = 0.154
*S* = 1.01
4674 reflections
226 parameters
H-atom parameters constrained
Δρ_max_ = 0.68 e Å^−3^
Δρ_min_ = −0.66 e Å^−3^

### 

Data collection: *APEX2* (Bruker, 2004 ▶); cell refinement: *SAINT* (Bruker, 2004 ▶); data reduction: *SAINT*; program(s) used to solve structure: *SHELXS97* (Sheldrick, 2008 ▶); program(s) used to refine structure: *SHELXL97* (Sheldrick, 2008 ▶); molecular graphics: *ORTEP-3* (Farrugia, 1997 ▶); software used to prepare material for publication: *SHELXL97* and *PLATON* (Spek, 2003 ▶).

## Supplementary Material

Crystal structure: contains datablocks I, global. DOI: 10.1107/S1600536808030985/ci2675sup1.cif

Structure factors: contains datablocks I. DOI: 10.1107/S1600536808030985/ci2675Isup2.hkl

Additional supplementary materials:  crystallographic information; 3D view; checkCIF report

## Figures and Tables

**Table 1:** Hydrogen-bond geometry (Å, °) *Cg*1 is the centroid of the C17–C22 ring.

*D*—H⋯*A*	*D*—H	H⋯*A*	*D*⋯*A*	*D*—H⋯*A*
C6—H6⋯O2^i^	0.98	2.46	3.426 (2)	168
C8—H8*B*⋯O2^i^	0.97	2.53	3.495 (3)	172
C21—H21⋯O1^ii^	0.93	2.53	3.248 (3)	134
C11—H11⋯*Cg*1^iii^	0.93	2.73	3.568 (2)	150

Symmetry codes: (i) 
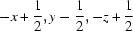
; (ii) 
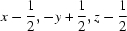
; (iii) 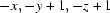.

**Fig. 1.**
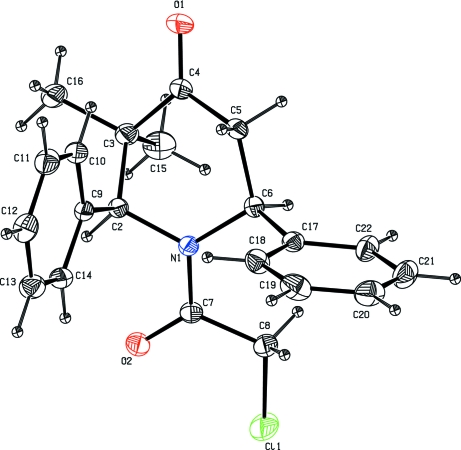
The molecular structure of the title compound, showing 20% probability displacement ellipsoids.

**Fig. 2.**
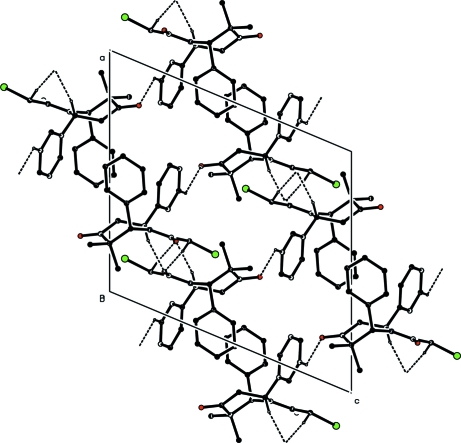
The crystal packing of the title compound, viewed along the b axis. Dashed lines indicate hydrogen bonds. H atoms not involed in hydrogen bonding have been omitted.

## supplementary crystallographic information

### Comment

Piperidones are the important group of heterocyclic compounds in the field of medicinal chemistry due to their biological activities, including cytotoxic properties (Dimmock *et al.*, 2001). They were also reported to possess analgesic, anti-inflammatory, central nervous system (CNS), local anaesthetic, anticancer and antimicrobial activities (Perumal *et al.*, 2001).

The sum of bond angles around N1 atom (359.4°) indicates *sp*^2^ hybridization. The N1—C7 [1.360 (2) Å] and C7—O2 [1.220 (2) Å] distances indicate electron delocalization. The piperidine ring adopts a distorted boat conformation, confirmed by puckering parameters q_2_ = 0.612 (2) Å, q_3_ = -0.122 (2) Å and φ_2_ = 258.0 (2)° (Cremer & Pople, 1975) and the asymmetry parameter Δ_s_(C2) = Δ_s_(C5) = 19.2 (2)° (Nardelli, 1983). The two phenyl rings are nearly orthogonal to each other with a dihedral angle of 87.1 (1)°. The methyl substituents are oriented equatorially [N1—C2—C3—C16 = 175.3 (2)°] and axially [N1—C2—C3—C15 = 55.1 (2)°] at C3 position.

The crystal structure is stabilized by C—H···O and C—H···π intermolecular interactions. The glide-related molecules are linked into a chain along the *b* axis by C6—H6···O2^i^ and C8—H8B···O2^i^ hydrogen bonds, and the chains are cross-linked *via* C21—H21···O1^ii^ hydrogen bonds; symmetry codes are given in Table 1. In addition, C—H···π interactions involving the C17–C22 ring (centroid *Cg*1) are observed.

### Experimental

A mixture of 3,3-dimethyl-*cis*-2,6-diphenylpiperidin-4-one (1.4 g, 5 mmol), chloro acetylchloride (0.8 ml, 5 mmol) and triethylamine (2 ml, 14.4 mmol) in anhydrous benzene (20 ml) was stirred at room temperature for 7 h. The benzene solution was dried over anhydrous Na_2_SO_4_ and concentrated to obtain a pasty mass. It was purified by crystallization from benzene–petroleum ether (95:5, 60–80°C).

### Refinement

H atoms were positioned geometrically (C—H = 0.93–0.98 Å) and allowed to ride on their parent atoms, with *U*_iso_(H) = 1.5*U*_eq_(C) for methyl H atoms and 1.2*U*_eq_(C) for other H atoms.

### Crystal data

**Table:**

C_21_H_22_ClNO_2_	*F*(000) = 752
*M**_r_* = 355.85	*D*_x_ = 1.272 Mg m^−^^3^
Monoclinic, *P*2_1_/*n*	Mo *K*α radiation, λ = 0.71073 Å
Hall symbol: -P 2yn	Cell parameters from 4674 reflections
*a* = 13.7005 (3) Å	θ = 2.5–28.5°
*b* = 9.8735 (2) Å	µ = 0.22 mm^−^^1^
*c* = 14.8960 (3) Å	*T* = 293 K
β = 112.762 (1)°	Block, colourless
*V* = 1858.08 (7) Å^3^	0.32 × 0.26 × 0.20 mm
*Z* = 4	

### Data collection

**Table:**

Bruker Kappa APEXII area-detector diffractometer	4674 independent reflections
Radiation source: fine-focus sealed tube	3424 reflections with *I* > 2σ(*I*)
graphite	*R*_int_ = 0.025
ω and φ scans	θ_max_ = 28.5°, θ_min_ = 2.5°
Absorption correction: multi-scan (SADABS; Sheldrick, 2001)	*h* = −18→18
*T*_min_ = 0.854, *T*_max_ = 0.958	*k* = −11→13
22063 measured reflections	*l* = −18→19

### Refinement

**Table:**

Refinement on *F*^2^	Primary atom site location: structure-invariant direct methods
Least-squares matrix: full	Secondary atom site location: difference Fourier map
*R*[*F*^2^ > 2σ(*F*^2^)] = 0.050	Hydrogen site location: inferred from neighbouring sites
*wR*(*F*^2^) = 0.154	H-atom parameters constrained
*S* = 1.01	*w* = 1/[σ^2^(*F*_o_^2^) + (0.0741*P*)^2^ + 0.7704*P*] where *P* = (*F*_o_^2^ + 2*F*_c_^2^)/3
4674 reflections	(Δ/σ)_max_ = 0.001
226 parameters	Δρ_max_ = 0.68 e Å^−^^3^
0 restraints	Δρ_min_ = −0.66 e Å^−^^3^

### Special details

**Table:**

Geometry. All e.s.d.'s (except the e.s.d. in the dihedral angle between two l.s. planes) are estimated using the full covariance matrix. The cell e.s.d.'s are taken into account individually in the estimation of e.s.d.'s in distances, angles and torsion angles; correlations between e.s.d.'s in cell parameters are only used when they are defined by crystal symmetry. An approximate (isotropic) treatment of cell e.s.d.'s is used for estimating e.s.d.'s involving l.s. planes.
Refinement. Refinement of *F*^2^ against ALL reflections. The weighted *R*-factor *wR* and goodness of fit *S* are based on *F*^2^, conventional *R*-factors *R* are based on *F*, with *F* set to zero for negative *F*^2^. The threshold expression of *F*^2^ > σ(*F*^2^) is used only for calculating *R*-factors(gt) *etc*. and is not relevant to the choice of reflections for refinement. *R*-factors based on *F*^2^ are statistically about twice as large as those based on *F*, and *R*- factors based on ALL data will be even larger.

### Fractional atomic coordinates and isotropic or equivalent isotropic displacement parameters (Å^2^)

**Table:**

	*x*	*y*	*z*	*U*_iso_*/*U*_eq_	
C2	0.20338 (13)	0.74492 (17)	0.41291 (11)	0.0364 (3)	
H2	0.2263	0.8253	0.3878	0.044*	
C3	0.30126 (14)	0.6958 (2)	0.50015 (13)	0.0457 (4)	
C4	0.27691 (14)	0.56295 (19)	0.53752 (13)	0.0460 (4)	
C5	0.19260 (15)	0.47683 (18)	0.46543 (13)	0.0450 (4)	
H5A	0.1275	0.4912	0.4755	0.054*	
H5B	0.2125	0.3828	0.4808	0.054*	
C6	0.16822 (13)	0.49786 (17)	0.35689 (12)	0.0374 (3)	
H6	0.2216	0.4486	0.3411	0.045*	
C7	0.16925 (13)	0.68513 (18)	0.24518 (12)	0.0392 (4)	
C8	0.15274 (18)	0.5752 (2)	0.16986 (14)	0.0536 (5)	
H8A	0.0834	0.5350	0.1537	0.064*	
H8B	0.2053	0.5048	0.1972	0.064*	
C9	0.10539 (13)	0.78759 (16)	0.43001 (11)	0.0372 (3)	
C10	0.08432 (16)	0.75494 (19)	0.51136 (13)	0.0462 (4)	
H10	0.1339	0.7062	0.5620	0.055*	
C11	−0.00972 (18)	0.7942 (2)	0.51791 (15)	0.0547 (5)	
H11	−0.0225	0.7717	0.5730	0.066*	
C12	−0.08371 (17)	0.8654 (2)	0.44463 (16)	0.0569 (5)	
H12	−0.1470	0.8906	0.4493	0.068*	
C13	−0.06398 (16)	0.8999 (2)	0.36330 (15)	0.0558 (5)	
H13	−0.1139	0.9490	0.3132	0.067*	
C14	0.02930 (15)	0.86157 (19)	0.35629 (13)	0.0451 (4)	
H14	0.0418	0.8855	0.3013	0.054*	
C15	0.39135 (16)	0.6647 (3)	0.46568 (19)	0.0695 (7)	
H15A	0.4103	0.7459	0.4409	0.104*	
H15B	0.3681	0.5975	0.4152	0.104*	
H15C	0.4517	0.6313	0.5194	0.104*	
C16	0.33953 (19)	0.8035 (2)	0.58046 (15)	0.0635 (6)	
H16A	0.3542	0.8862	0.5541	0.095*	
H16B	0.4028	0.7724	0.6321	0.095*	
H16C	0.2856	0.8194	0.6054	0.095*	
C17	0.06144 (13)	0.43346 (18)	0.30018 (12)	0.0402 (4)	
C18	−0.03188 (15)	0.4922 (2)	0.29576 (15)	0.0514 (5)	
H18	−0.0308	0.5772	0.3230	0.062*	
C19	−0.12687 (16)	0.4244 (3)	0.25075 (18)	0.0653 (6)	
H19	−0.1896	0.4643	0.2477	0.078*	
C20	−0.12916 (18)	0.2988 (3)	0.21055 (17)	0.0672 (6)	
H20	−0.1932	0.2534	0.1810	0.081*	
C21	−0.0373 (2)	0.2404 (2)	0.21401 (17)	0.0645 (6)	
H21	−0.0389	0.1556	0.1862	0.077*	
C22	0.05803 (17)	0.3072 (2)	0.25877 (14)	0.0517 (5)	
H22	0.1203	0.2669	0.2611	0.062*	
Cl1	0.16240 (6)	0.63835 (7)	0.06314 (4)	0.0778 (2)	
N1	0.17457 (10)	0.64277 (14)	0.33383 (9)	0.0350 (3)	
O1	0.32116 (13)	0.52655 (16)	0.62105 (10)	0.0706 (5)	
O2	0.17704 (12)	0.80385 (14)	0.22634 (10)	0.0518 (3)	

### Atomic displacement parameters (Å^2^)

**Table:**

	*U*^11^	*U*^22^	*U*^33^	*U*^12^	*U*^13^	*U*^23^
C2	0.0408 (8)	0.0334 (8)	0.0325 (8)	−0.0040 (6)	0.0113 (6)	0.0006 (6)
C3	0.0407 (8)	0.0456 (10)	0.0409 (9)	−0.0039 (7)	0.0049 (7)	0.0007 (7)
C4	0.0477 (9)	0.0416 (10)	0.0396 (9)	0.0071 (8)	0.0070 (7)	0.0041 (7)
C5	0.0533 (10)	0.0358 (9)	0.0399 (9)	−0.0014 (7)	0.0114 (8)	0.0063 (7)
C6	0.0398 (8)	0.0331 (8)	0.0384 (8)	−0.0020 (6)	0.0143 (7)	0.0000 (6)
C7	0.0394 (8)	0.0433 (10)	0.0375 (8)	−0.0005 (7)	0.0177 (7)	0.0022 (7)
C8	0.0761 (13)	0.0507 (11)	0.0404 (9)	0.0006 (10)	0.0295 (9)	0.0007 (8)
C9	0.0452 (8)	0.0321 (8)	0.0328 (8)	−0.0034 (7)	0.0136 (6)	−0.0017 (6)
C10	0.0599 (10)	0.0423 (10)	0.0382 (9)	−0.0002 (8)	0.0210 (8)	0.0036 (7)
C11	0.0692 (12)	0.0547 (12)	0.0506 (11)	−0.0083 (10)	0.0346 (10)	−0.0026 (9)
C12	0.0509 (10)	0.0616 (13)	0.0633 (13)	−0.0042 (9)	0.0278 (10)	−0.0119 (10)
C13	0.0501 (10)	0.0628 (13)	0.0487 (11)	0.0097 (9)	0.0127 (8)	−0.0009 (9)
C14	0.0521 (10)	0.0471 (10)	0.0356 (8)	0.0046 (8)	0.0164 (7)	0.0025 (7)
C15	0.0394 (10)	0.0911 (18)	0.0705 (15)	0.0000 (10)	0.0129 (10)	0.0047 (13)
C16	0.0668 (13)	0.0553 (12)	0.0468 (11)	−0.0150 (10)	−0.0016 (9)	−0.0027 (9)
C17	0.0453 (9)	0.0390 (9)	0.0351 (8)	−0.0074 (7)	0.0144 (7)	0.0031 (7)
C18	0.0458 (9)	0.0456 (10)	0.0566 (11)	−0.0039 (8)	0.0129 (8)	0.0041 (9)
C19	0.0424 (10)	0.0691 (15)	0.0728 (15)	−0.0048 (10)	0.0096 (10)	0.0161 (12)
C20	0.0586 (12)	0.0710 (15)	0.0568 (12)	−0.0287 (11)	0.0057 (10)	0.0037 (11)
C21	0.0760 (15)	0.0581 (13)	0.0567 (12)	−0.0258 (11)	0.0225 (11)	−0.0140 (10)
C22	0.0605 (11)	0.0497 (11)	0.0489 (10)	−0.0143 (9)	0.0256 (9)	−0.0101 (8)
Cl1	0.1234 (6)	0.0747 (4)	0.0530 (3)	−0.0077 (4)	0.0534 (4)	−0.0031 (3)
N1	0.0387 (7)	0.0337 (7)	0.0327 (7)	−0.0025 (5)	0.0139 (5)	0.0002 (5)
O1	0.0861 (11)	0.0565 (9)	0.0427 (8)	0.0064 (8)	−0.0041 (7)	0.0112 (7)
O2	0.0714 (9)	0.0441 (7)	0.0471 (7)	−0.0043 (6)	0.0308 (7)	0.0057 (6)

### Geometric parameters (Å, °)

**Table:**

C2—N1	1.483 (2)	C11—C12	1.363 (3)
C2—C9	1.520 (2)	C11—H11	0.93
C2—C3	1.541 (2)	C12—C13	1.382 (3)
C2—H2	0.98	C12—H12	0.93
C3—C4	1.512 (3)	C13—C14	1.375 (3)
C3—C16	1.534 (3)	C13—H13	0.93
C3—C15	1.540 (3)	C14—H14	0.93
C4—O1	1.209 (2)	C15—H15A	0.96
C4—C5	1.500 (3)	C15—H15B	0.96
C5—C6	1.533 (2)	C15—H15C	0.96
C5—H5A	0.97	C16—H16A	0.96
C5—H5B	0.97	C16—H16B	0.96
C6—N1	1.482 (2)	C16—H16C	0.96
C6—C17	1.517 (2)	C17—C18	1.382 (3)
C6—H6	0.98	C17—C22	1.384 (3)
C7—O2	1.220 (2)	C18—C19	1.384 (3)
C7—N1	1.360 (2)	C18—H18	0.93
C7—C8	1.514 (3)	C19—C20	1.372 (4)
C8—Cl1	1.7595 (19)	C19—H19	0.93
C8—H8A	0.97	C20—C21	1.367 (4)
C8—H8B	0.97	C20—H20	0.93
C9—C10	1.388 (2)	C21—C22	1.382 (3)
C9—C14	1.393 (2)	C21—H21	0.93
C10—C11	1.385 (3)	C22—H22	0.93
C10—H10	0.93		
			
N1—C2—C9	109.92 (12)	C10—C11—H11	119.6
N1—C2—C3	109.45 (14)	C11—C12—C13	119.48 (19)
C9—C2—C3	118.87 (14)	C11—C12—H12	120.3
N1—C2—H2	105.9	C13—C12—H12	120.3
C9—C2—H2	105.9	C14—C13—C12	120.11 (19)
C3—C2—H2	105.9	C14—C13—H13	119.9
C4—C3—C16	111.85 (16)	C12—C13—H13	119.9
C4—C3—C15	105.63 (18)	C13—C14—C9	121.22 (18)
C16—C3—C15	108.79 (18)	C13—C14—H14	119.4
C4—C3—C2	109.86 (14)	C9—C14—H14	119.4
C16—C3—C2	111.27 (16)	C3—C15—H15A	109.5
C15—C3—C2	109.26 (16)	C3—C15—H15B	109.5
O1—C4—C5	120.59 (18)	H15A—C15—H15B	109.5
O1—C4—C3	122.87 (17)	C3—C15—H15C	109.5
C5—C4—C3	116.54 (15)	H15A—C15—H15C	109.5
C4—C5—C6	118.12 (15)	H15B—C15—H15C	109.5
C4—C5—H5A	107.8	C3—C16—H16A	109.5
C6—C5—H5A	107.8	C3—C16—H16B	109.5
C4—C5—H5B	107.8	H16A—C16—H16B	109.5
C6—C5—H5B	107.8	C3—C16—H16C	109.5
H5A—C5—H5B	107.1	H16A—C16—H16C	109.5
N1—C6—C17	113.96 (13)	H16B—C16—H16C	109.5
N1—C6—C5	111.52 (14)	C18—C17—C22	119.07 (17)
C17—C6—C5	107.46 (13)	C18—C17—C6	121.70 (16)
N1—C6—H6	107.9	C22—C17—C6	119.00 (16)
C17—C6—H6	107.9	C17—C18—C19	119.9 (2)
C5—C6—H6	107.9	C17—C18—H18	120.0
O2—C7—N1	122.85 (16)	C19—C18—H18	120.0
O2—C7—C8	121.32 (16)	C20—C19—C18	120.4 (2)
N1—C7—C8	115.83 (15)	C20—C19—H19	119.8
C7—C8—Cl1	111.96 (14)	C18—C19—H19	119.8
C7—C8—H8A	109.2	C21—C20—C19	120.0 (2)
Cl1—C8—H8A	109.2	C21—C20—H20	120.0
C7—C8—H8B	109.2	C19—C20—H20	120.0
Cl1—C8—H8B	109.2	C20—C21—C22	120.1 (2)
H8A—C8—H8B	107.9	C20—C21—H21	120.0
C10—C9—C14	117.72 (16)	C22—C21—H21	120.0
C10—C9—C2	125.29 (15)	C21—C22—C17	120.5 (2)
C14—C9—C2	116.96 (15)	C21—C22—H22	119.8
C11—C10—C9	120.67 (18)	C17—C22—H22	119.8
C11—C10—H10	119.7	C7—N1—C6	122.42 (14)
C9—C10—H10	119.7	C7—N1—C2	117.37 (13)
C12—C11—C10	120.80 (18)	C6—N1—C2	119.59 (13)
C12—C11—H11	119.6		
			
N1—C2—C3—C4	−60.29 (18)	C12—C13—C14—C9	0.2 (3)
C9—C2—C3—C4	67.1 (2)	C10—C9—C14—C13	−0.6 (3)
N1—C2—C3—C16	175.29 (15)	C2—C9—C14—C13	177.27 (17)
C9—C2—C3—C16	−57.3 (2)	N1—C6—C17—C18	−50.6 (2)
N1—C2—C3—C15	55.1 (2)	C5—C6—C17—C18	73.5 (2)
C9—C2—C3—C15	−177.45 (17)	N1—C6—C17—C22	134.95 (16)
C16—C3—C4—O1	−28.9 (3)	C5—C6—C17—C22	−100.95 (19)
C15—C3—C4—O1	89.3 (2)	C22—C17—C18—C19	0.3 (3)
C2—C3—C4—O1	−153.0 (2)	C6—C17—C18—C19	−174.15 (18)
C16—C3—C4—C5	150.31 (18)	C17—C18—C19—C20	0.2 (3)
C15—C3—C4—C5	−91.50 (19)	C18—C19—C20—C21	−0.6 (4)
C2—C3—C4—C5	26.2 (2)	C19—C20—C21—C22	0.6 (4)
O1—C4—C5—C6	−157.96 (19)	C20—C21—C22—C17	−0.2 (3)
C3—C4—C5—C6	22.8 (2)	C18—C17—C22—C21	−0.3 (3)
C4—C5—C6—N1	−37.4 (2)	C6—C17—C22—C21	174.29 (18)
C4—C5—C6—C17	−162.92 (16)	O2—C7—N1—C6	−178.13 (16)
O2—C7—C8—Cl1	7.0 (2)	C8—C7—N1—C6	1.9 (2)
N1—C7—C8—Cl1	−173.12 (13)	O2—C7—N1—C2	−7.2 (2)
N1—C2—C9—C10	109.58 (18)	C8—C7—N1—C2	172.90 (15)
C3—C2—C9—C10	−17.6 (2)	C17—C6—N1—C7	−66.5 (2)
N1—C2—C9—C14	−68.14 (18)	C5—C6—N1—C7	171.59 (15)
C3—C2—C9—C14	164.68 (16)	C17—C6—N1—C2	122.72 (15)
C14—C9—C10—C11	0.5 (3)	C5—C6—N1—C2	0.8 (2)
C2—C9—C10—C11	−177.24 (17)	C9—C2—N1—C7	104.00 (16)
C9—C10—C11—C12	0.2 (3)	C3—C2—N1—C7	−123.72 (15)
C10—C11—C12—C13	−0.6 (3)	C9—C2—N1—C6	−84.78 (17)
C11—C12—C13—C14	0.5 (3)	C3—C2—N1—C6	47.50 (18)

### Hydrogen-bond geometry (Å, °)

**Table:**

*D*—H···*A*	*D*—H	H···*A*	*D*···*A*	*D*—H···*A*
C6—H6···O2^i^	0.98	2.46	3.426 (2)	168
C8—H8B···O2^i^	0.97	2.53	3.495 (3)	172
C21—H21···O1^ii^	0.93	2.53	3.248 (3)	134
C11—H11···Cg1^iii^	0.93	2.73	3.568 (2)	150

Symmetry codes: (i) −*x*+1/2, *y*−1/2, −*z*+1/2; (ii) *x*−1/2, −*y*+1/2, *z*−1/2; (iii) −*x*, −*y*+1, −*z*+1.
